# *Streptococcus pneumoniae* and *Haemophilus influenzae* type b Carriage, Central Asia

**DOI:** 10.3201/eid1109.040798

**Published:** 2005-09

**Authors:** Stephanie H. Factor, Leslye LaClaire, Melinda Bronsdon, Fleura Suleymanova, Gulbanu Altynbaeva, Bakhtiyar A. Kadirov, Uulkan Shamieva, Scott F. Dowell, Anne Schuchat, Richard Facklam, Benjamin Schwartz, Terence Chorba

**Affiliations:** * Centers for Disease Control and Prevention, Atlanta, Georgia, USA;; † New York City Department of Health and Mental Hygiene, New York, New York, USA;; ‡ Zhambyl Oblast Children's Infectious Disease Hospital, Taraz City, Kazakhstan;; § Uzbekistan Ministry of Health, Tashkent, Uzbekistan;; ¶ Osh Oblast Children's Infectious Diseases Hospital, Osh, Kyrgyz Republic;; # Thai Ministry of Public Health, Bangkok, Thailand

**Keywords:** Streptococcus pneumoniae, Haemophilus influenzae, nasopharyngeal carriage, swab study, Central Asian Republics, Kazakhstan, Kyrgyz Republic, Uzbekistan, pneumococcal-conjugate vaccine, Hib-conjugate vaccine, dispatch

## Abstract

A study of children was conducted in 3 Central Asian Republics. Approximately half of the *Streptococcus pneumoniae* isolates were serotypes included in available vaccine formulations. Approximately 6% of children carried *Haemophilus influenzae* type b (Hib). Using pneumococcal and Hib conjugate vaccines may decrease illness in the Central Asian Republics.

*Streptococcus pneumoniae* and *Haemophilus influenzae* cause a large percentage of acute respiratory and invasive bacterial infections throughout the world (*1*). Acute respiratory infection is the leading cause of childhood death in the Central Asian Republics of the former Soviet Union (*2**,**3*), a region that includes Kazakhstan, Uzbekistan, Turkmenistan, Tajikistan, and the Kyrgyz Republic. These deaths occur despite the availability and use of antimicrobial drugs throughout the former Soviet Union (*4**,**5*).

To prevent illness from *S. pneumoniae* in the United States, the 7-valent pneumococcal conjugate vaccine (Prevnar, Wyeth Pharmaceuticals, Philadelphia, PA, USA) was added to the routine infant immunization schedule in 2000. Prevnar contains *S. pneumoniae* serotypes 4, 6B, 9V, 14, 18C, 19F, and 23F. Higher valency formulations (9-, 11-, and 13-valent) are under evaluation. The 9-valent formulation (including types 1 and 5) was successful in South Africa (*6*) and The Gambia (*7*), and an 11-valent formulation (including types 1, 3, 5, and 7F) is being studied in the Philippines. An accelerated development and introduction plan for pneumococcal conjugate vaccines for use in developing countries is supported by the Global Alliance for Vaccines and Immunization (http://www.preventpneumonia.com).

*H. influenzae* type b (Hib) conjugate vaccines have been recommended for infants in the United States since 1990. Widespread use of these vaccines has dramatically reduced Hib invasive disease in both industrialized and developing countries (*8**,**9*). The World Health Organization (WHO) has recommended use of the Hib conjugate vaccine in regions of the world where the extent of Hib disease has been established. Prevalence of Hib invasive disease must be assessed in countries in the Central Asian Republics before introducing the Hib conjugate vaccine.

Laboratory data to determine prevalence of *S. pneumoniae* and Hib are not collected in the Central Asian Republics. To determine the benefits of using the pneumococcal and Hib conjugate vaccines in these countries, we conducted a nasopharyngeal swab survey of pediatric patients to identify the most prevalent serotypes and penicillin-resistance patterns of *S. pneumoniae* and to assess the presence of Hib.

## The Study

In January 1997, we obtained nasopharyngeal swabs from a convenience sample of both ill and well children, ages 2–59 months, who were visiting outpatient clinics in Taraz City (formerly Djambul), Kazakhstan; Fergana, Uzbekistan; and Osh, Kyrgyz Republic. Before swabs were obtained, written parental consent was obtained in Russian, Kazak, Kyrgyz, or Uzbek under a protocol approved by a local institutional review board and the Centers for Disease Control and Prevention (CDC).

Nasopharyngeal swab collection and pathogen isolation have been described previously (*1*). Briefly, a flexible calcium alginate swab was inserted through the nares to the nasopharynx, rotated ≈180°, and withdrawn. While in the field, the swabs were first streaked on chocolate agar (CA) plates containing bacitracin to isolate *H. influenzae*, and then onto Trypticase soy 5% sheep blood agar plates containing gentamicin to isolate *S. pneumoniae*. All plates were brought back to the laboratory and incubated appropriately. Pure *H. influenzae* cultures were isolated and spread onto quad plates. Those colonies that grew on only the XV and blood quadrants were considered to be *H. influenzae* and were saved on CA slants. Suspected *S. pneumoniae* colonies were streaked onto conventional 5% sheep blood agar plates with an optochin disk added. After appropriate incubation, α-hemolytic isolates with an optochin inhibition zone >14 mm were considered to be *S. pneumoniae* and saved on CA slants. CA slants of both *H. influenzae* and *S. pneumoniae* were transported to CDC in Atlanta. Isolates of *H. influenzae* were serotyped with Difco *H. influenzae* serotype-specific rabbit antisera (BD, Sparks, MD, USA), and *S. pneumoniae* isolates were serotyped with CDC-prepared antiserum. *S. pneumoniae* cultures were tested for antimicrobial susceptibility to penicillin with broth dilution MIC testing by using the guidelines of the Clinical and Laboratory Standards Institute (formerly NCCLS) and customized MIC panels.

Results were similar in all 3 sites, so data were combined. The method of isolate storage and transport resulted in different survival rates among isolates (Tables 1 and 2). Low rates of *S. pneumoniae* isolates among children receiving antimicrobial drugs prevent any conclusions about that group. Among *S. pneumoniae* and *H. influenzae* isolates, survival was negatively associated with duration of storage. Among *S. pneumoniae* isolates, survival was positively associated with increasing age. However, the lack of any trends in Hib colonization and *S. pneumoniae* nonsusceptibility by age and duration of storage suggests that differential survival did not produce bias.

**Table 1:** *Streptococcus pneumoniae* in convenience sample, Central Asian Republics, January 1997*

Variable	% SP colonization (n/N)	% SP isolate survival (n/N)	% SP PCN nonsusceptible isolates (n/N)	Calculated % colonization with PCN-nonsusceptible SP†
Age (mo)				
2–5	49 (47/95)	49 (23/47)	17 (4/23)	8
6–11	64 (74/115)	54 (40/74)	38 (15/40)	24
12–23	66 (94/142)	60 (56/94)	27 (15/56)	18
24–35	62 (65/105)	62 (40/65)	23 (9/40)	14
36–47	58 (61/106)	64 (39/61)	18 (7/39)	10
48–59	51 (34/67)	79 (27/34)	19 (5/27)	10
Sex				
Male	60 (197/331)	60 (119/197)	27 (32/119)	16
Female	60 (178/299)	60 (106/178)	22 (22/106)	13
Reported use of antimicrobial drugs in past 7 days				
Yes	49 (34/70)	29 (10/34)	10 (1/10)	5
No	61 (335/552)	64 (213/335)	24 (52/213)	15
Weeks storage before transport				
3	65 (72/110)	25 (18/72)	11 (2/18)	7
2	56 (175/315)	62 (108/175)	24 (27/108)	13
1	62 (128/205)	77 (99/128)	26 (26/99)	16
Total	59 (375/630)	60 (225/375)	24 (55/225)	14

*SP, *S. pneumoniae*; PCN, penicillin. †Result obtained by multiplying the percentage of children colonized with SP times the percentage of SP isolates that are nonsusceptible to penicillin (percentages in column 1 multiplied by the percentages in column 3).

**Table 2:** *Haemophilus influenzae* in convenience sample, Central Asian Republics, January 1997*

Variable	% HI colonization (n/N)	% HI isolate survival (n/N)	% Hib among all HI isolates (n/N)	Calculated % colonization with Hib†
Age (mo)				
2–5	45 (43/95)	77 (33/43)	6 (2/33)	3
6–11	59 (68/115)	76 (52/68)	17 (9/52)	10
12–23	60 (85/142)	89 (76/85)	8 (6/76)	5
24–35	60 (63/105)	87 (55/63)	13 (7/55)	8
36–47	58 (62/106)	85 (53/62)	13 (7/53)	4
48–59	54 (36/67)	86 (31/36)	10 (3/31)	5
Sex				
Male	57 (187/331)	82 (154/187)	13 (20/154)	7
Female	57 (170/299)	86 (146/170)	10 (14/146)	8
Reported use of antimicrobial drugs in past 7 days				
Yes	57 (40/70)	82 (33/40)	9 (3/33)	5
No	56 (310/552)	84 (260/310)	12 (31/260)	7
Weeks storage before transport				
3	59 (65/110)	71 (46/65)	20 (9/46)	12
2	57 (178/315)	83 (147/178)	10 (14/147)	6
1	56 (114/205)	94 (107/114)	10 (11/107)	6
Total	57 (357/630)	84 (300/357)	11 (34/300)	6

*HI, *Haemophilus influenzae*; Hib, *H. influenzae* type b. †Result obtained by multiplying percentage of children colonized with HI times the proportion of Hib isolates (percentages in column 1 multiplied by percentages in column 3).

Of 630 children swabbed, 375 (59%) were colonized with *S. pneumoniae*. Of the 375 isolates, 224 S. pneumoniae isolates were available for susceptibility testing and serotyping. Of the 224 isolates, 54 (24%) were nonsusceptible to penicillin. The 9 most common serotypes in decreasing order were 19F (17% of isolates), 6B (15%), 6A (9%), 14 (6%), 23B (4%), 19A (3%), 23F (3%), 18C (2%), and 4 (2%). These accounted for 61% of all isolates.

In our sample, the 7-valent pneumococcal conjugate vaccine would cover 47% of pneumococcal isolates, the 9-valent would cover 48%, and the 11-valent would cover 51%. Of all the serotypes covered in these vaccines, serotypes 6B, 14, 19F, and 23F account for all nonsusceptible strains. Because all 3 vaccines contain these 4 serotypes, each vaccine would cover 33 (61%) of 54 nonsusceptible isolates of *S. pneumoniae*. An additional 13% of nonsusceptible strains are vaccine-related (strains 6A [4 of 54, 7%] and 23B [3 of 54, 6%]).

Of the 630 children from whom nasopharyngeal swabs were obtained, 357 (57%) were carrying *H. influenzae*. Of the 300 isolates available for serotyping, 34 (11%) were Hib. When Hib carriage is determined by multiplying the percentage of children colonized with *H. influenzae* times the percentage of Hib among all *H. influenzae* isolates tested, the carriage rate is 6% (Table 2).

## Conclusions

Our survey showed that most children in these Central Asian Republics were colonized with at least 1 potential respiratory pathogen. Approximately half of the *S. pneumoniae* isolates and more than half of the penicillin-nonsusceptible *S. pneumoniae* isolates are included in the available pneumococcal conjugate vaccine formulations. Approximately 6% of the children in this convenience sample were carrying Hib.

The colonization rate of Hib found in our study is similar to rates observed in industrialized populations before Hib conjugate vaccines were widely used. Carriage rates for Hib before widespread vaccination in Finland, the United Kingdom, and the United States were 2%–6% (*10**–**13*). In these countries, introduction of the Hib vaccine virtually eliminated Hib invasive disease (*13*).

Assessing the prevalence of disease due to specific respiratory pathogens is difficult; blood cultures are insensitive, and other diagnostic tests are not specific. Nasopharyngeal colonization surveys of groups of children identify the predominant organisms circulating in the community and the presence or absence of antimicrobial-drug resistance. The presence of *S. pneumoniae* serotypes found in the pneumococcal conjugate vaccine suggests this vaccine may decrease some illness from acute respiratory infection. The experience in other countries with similar prevaccination Hib nasopharyngeal carriage rates suggests that the Hib conjugate vaccine may also decrease illness. These findings may be helpful in the decision-making process regarding the value of introducing conjugate vaccines for Hib and pneumococcal disease prevention.
